# Application of IoT Authentication Key Management Algorithm to Personnel Information Management

**DOI:** 10.1155/2022/4584072

**Published:** 2022-04-21

**Authors:** Qing Wang, Haoran Li

**Affiliations:** Shandong Youth University of Political Science, Jinan, Shandong 250103, China

## Abstract

A new IoT authentication protocol is presented to address the security deficiencies in the Z-Wave protocol. The new protocol is based on Diameter and includes authentication/authorization module, billing module, and secure communication module. According to the characteristics of IoT devices, the relevant algorithms are optimized, and the key agreement scheme based on elliptic curve algorithm and the symmetric encryption scheme based on AES and RC4 are introduced, which enhances the security of the protocol and also improves the system performance. The speed of response reduces the energy consumption of the system. Aiming at solving the problem that the existing polynomial-based key predistribution management scheme is limited by the key sharing rate between the nodes and the network connectivity rate, a quadratic-based wireless sensor key management scheme is proposed. The scheme breaks through the existing idea of building a shared key with a binary t-order symmetric polynomial, introduces a multivariate asymmetric quadratic polynomial, and utilizes the relationship between the quadratic eigenvalues and eigenvectors. The analysis proves that the quadratic form can be orthogonally diagonalized and uses it to generate key information, and nodes realize identity authentication by exchanging key information. The establishment of an independent and unique session key with the neighbor node is completed. The security performance analysis and simulation results show that, compared with the existing key management schemes, the scheme has great improvements in anti-capture property, connectivity, scalability, communication overhead, and storage overhead. After a series of functional tests, the enterprise information system based on the SaaS platform in this paper basically met the design requirements and finally realized the networking of the enterprise information management process and the sharing of information. Each functional module of the system can be used normally. When the input and output are wrong, the system will have a correct prompt. The buttons and various controls of the system can work normally, meeting the requirements of functional testing. Each document of the system is correct and complete, and the language description and logic meet the needs of users and meet the requirements of document testing. The test results show that the interface of the system is friendly and easy to operate and the performance of the system is stable, which is basically in line with the needs of users and achieves the design goal of this system.

## 1. Introduction

Key management is an important part of keeping IoT secure. As the foundation of secure cryptography, key management plays an important role in cryptography-based IoT security solutions. The reason for this is that, in the current cryptography, all cryptographic algorithms required to be designed can be publicly evaluated; that is, the security of the entire cryptosystem does not depend on the secrecy of the cryptographic algorithm or the protection of the encryption device, but on the encryption of the password [1]. It can be seen that the management of keys is the most worthy of attention in any cryptographic algorithm or even cryptosystem. The Internet of Things is composed of a variety of heterogeneous networks, such as sensor networks at the perception layer and the Internet at the network layer [2, 3].

Modern society is an information-based society. With the development of science and technology, the globalization of the economy has made the competition among enterprises fiercer and fiercer [4]. For the development of enterprises, informatization is an urgent need to solve the survival and development of enterprises and enhance their international competitiveness [5]. Modern enterprise management not only is the management of people, finances, and things, but has developed into a comprehensive resource management of people, finances, things, and information. Whether these resources are used well or not has a direct impact on the business performance of an enterprise [6]. Enterprise resource planning (ERP), as a new enterprise management idea, has become a hot spot in today's business world. The successful realization of enterprise resource planning (ERP) must be based on a very stable and high-performance network, and the requirements for network security in the realization of ERP are essential. If the network does not meet the required security level, the advantages of ERP cannot be fully exerted, and even the implementation effect of the ERP system in the enterprise will be seriously affected [7]. Therefore, ensuring the security of network and data is one of the basic requirements of enterprise information network construction. The construction of enterprise network security should not only ensure the security of the enterprise local area network, but also consider the security of networking with partners, suppliers, and branch offices; it is necessary to realize information sharing within the local area network of the organization and also consider the security of various departments. It is necessary to strengthen network security without significantly reducing network performance.

With the rapid development of economic construction, the modernization of management disciplines and management methods has been mentioned in a very important position [8]. The application of management information systems based on computer processing is the development direction of modern enterprise management. The management information system is based on the three aspects of management theory and practice, computer information technology, and management organization and develops with the development of these three aspects. At present, the world's more advanced enterprise management information system, ERP, has been widely used and is increasingly valued by the business community [9]. With the acceleration of network globalization, the popularization of information technology and the construction of information superhighway have created a good foundation for the development and sharing of information resources. Information is the most important resource in the 21st century, and the security of information has naturally become a topic of common concern to the whole society. The information society has put forward higher and higher requirements for the construction of information infrastructure; the development of information resources; and the speed, accuracy, and comprehensiveness of information services, especially the security of information [10]. The importance of information has finally reached a level of qualitative change in the process of human civilization. The society is using information more and more widely, and its dependence on information is also deepening. At the same time, the security issues of information and information systems will inevitably become more and more important. The use of external resources after the integration of internal resources makes ERP gradually expand to the network, so the security issues of ERP systems along with the security issues of the network are increasingly attracting more and more people's attention. As a management information system, ERP system is bound to face the security problems commonly faced by information systems, and at the same time, it also has special security requirements determined by its own characteristics. Therefore, the research on the security of ERP system has very important theoretical and practical significance.

This paper makes a detailed design of the introduced Diameter-based IoT authentication scheme. The message format of the scheme is introduced, and the Command Class and Command used in Z-Wave are introduced. The detailed design process of key management and authentication accounting is given. Key management includes key negotiation and key update. Key negotiation ensures that both parties in the communication can obtain the same key safely, and key update ensures that the security of the system will not be weakened over time. In terms of authentication, the authentication process for nodes joining the network is introduced in detail. In the transmission of Z-Wave plaintext, the security of data and the legitimacy of nodes are guaranteed. In this paper, a quadratic-based wireless sensor key management scheme is proposed. This scheme breaks through the existing idea of establishing a shared key with a binary t-degree symmetric polynomial, introduces a multivariate asymmetric quadratic multiform, and uses the quadratic orthogonality. Diagonalization is used to establish session keys, and a new idea based on multivariate polynomial key predistribution is proposed. Security performance analysis and simulation show that, compared with the existing key management schemes, the proposed scheme has great improvements in anti-capture property, connectivity, scalability, communication overhead, and storage overhead. Under the guidance of SaaS design method, the enterprise information system is constructed using SaaS development tools. In the process of implementation, the requirements of enterprise information management system are first studied. On this basis, the application of SaaS multitenant single instance is designed.

## 2. Related Work

The main goals of IoT security are network availability, controllability, and confidentiality; integrity; authenticity; identifiability; and freshness of information [11]. The components of the Internet of Things include sensor devices, transmission systems, and processing systems, which are structurally located in the perception layer, network layer, processing layer, and application layer of the Internet of Things. Correspondingly, its security forms are node security, network and information system security, and information processing security. Node security corresponds to the security of the perception layer of the Internet of Things. The perception layer of the Internet of Things consists of sensors, RFID, and other perception terminals. The corresponding security includes the physical security of nodes and the security of information collection. The network layer is the information backbone of the Internet of Things. Information transmission security corresponds to the network layer security of the Internet of Things: ensuring the confidentiality, integrity, authenticity, and freshness of data in the process of information transmission, mainly the security of the communication network, involving secure routing [12].

Relevant scholars proposed the first random key predistribution protocol, EG protocol [13]. According to the random graph theory, the protocol uses the shared key probability between nodes to ensure the existence of a shared key between nodes. The protocol includes shared key discovery, key establishment, key management process for key revocation, restart key, and dynamically adding nodes. However, the security of this protocol is not high, the use of keys in nodes is insufficient, and the amount of key storage is large [14]. The researchers improved the EG protocol and proposed a q-composite random key predistribution protocol, establishing a random key pool *S*; each node randomly selects and stores *m* keys from the key pool *S*, and each node must discover all shared keys with its neighbors [15]. After the key discovery phase, each node shares at least *q* keys with its neighbors, which ensures that there are at least *q* shared keys between two nodes with probability *p*. The q-composite protocol has better capture resistance when the number of captured nodes is small, and when a large number of nodes are captured, its capture resistance becomes poor.

According to the deployment of sensor nodes, a key predistribution protocol based on node deployment knowledge is proposed [16]. The protocol uses a quadrilateral format to describe the deployment geographical situation and distributes node keys, avoiding unnecessary key distribution and saving storage. The researchers propose a key predistribution protocol based on the location information of the deployment area [17]. The network deployment is divided into quadrilateral areas, and the key pool is also divided into subsets corresponding to the areas and then deployed according to the divided areas. The probability of sharing keys between nodes in the same area is improved, and the nodes between adjacent areas also increase the probability of sharing keys through the overlap of key subsets, thereby improving the connectivity of the network and reducing the required number of nodes [18].

Relevant scholars pointed out that in the key management scheme based on symmetric cryptosystem, the simplest method is to share a key among all nodes in the whole network, that is, to load the same key in each node [19]. This scheme has some advantages such as no dependence on base station, low computational complexity, and easy management. However, the scheme has serious security problems. Once the attacker obtains the key of a node, the attacker controls the nodes in the entire network; another relatively secure scheme is to assign the same key to each pair of nodes in the network. The node communicates using the key shared with the peer node. The scheme not only has the advantage of not relying on the base station, but also will not affect the security of other nodes in the network [20]. However, the scalability of the scheme is poor, and it will be difficult to add new nodes to the network. The nodes in the scheme need to store more keys.

## 3. Method

### 3.1. Overall Structure of the Scheme

This solution is based on the Diameter protocol and introduces a new Z-Wave application layer protocol to provide end-to-end security. With this solution, end-to-end security between nodes can be provided under the Z-Wave protocol. Moreover, a new key negotiation and management scheme is introduced, which avoids the one-time pad scheme in the secure communication process of the Z-Wave protocol, which can effectively improve the performance of the Z-Wave protocol. The main features of this program are as follows:

1. The data format of the Diameter protocol is adopted: messages and AVP. It has excellent flexibility and extensibility, and based on the Diameter basic protocol and NAS extension applications, new messages and AVPs are introduced to meet some features and processes of the new protocol.
2. On the IoT node device and authentication platform, the TCP network security communication such as IPsec and TLS secure communication used in the underlying communication of the Diameter protocol is abandoned. In this scheme, AES and RC4 are used to encrypt data of different lengths. Many of the newly added messages and AVPs are used for new secure communications.
3. A new key distribution and management scheme is introduced. Key distribution plays a very important role in the process of the platform starting up, nodes joining the network, and the platform re-requesting authentication. This is essential for a complete authentication protocol. Starting from low power consumption and fast calculation, this scheme introduces ECC-based digital signature algorithm (ECDSA) and key agreement ECDH algorithm to securely and quickly perform key negotiation and distribution.
4. As an application of the Z-Wave protocol, this scheme is used to authenticate, bill, and ensure the end-to-end secure communication of the Z-Wave nodes. All data packets of this protocol belong to a Command Class of the Z-Wave protocol. Z-Wave nodes join the Z-Wave network as nonsecure devices for authentication, billing, and communication through this protocol. In some scenarios where performance is not a concern, overall security can be further enhanced by incorporating security devices into the Z-Wave network.

**Figure 1:**
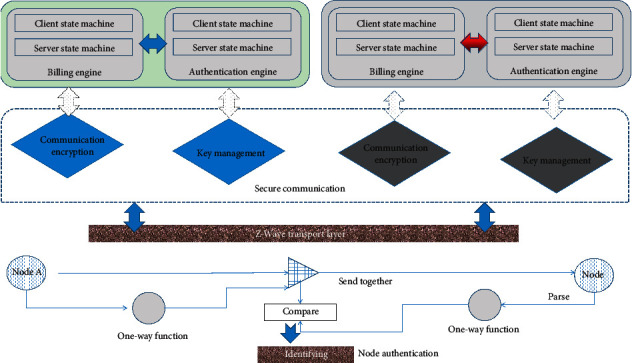
Overall structure of the protocol.

The overall structure of this scheme is shown in Figure 1. Platforms and nodes can choose to implement only the billing function or authentication function according to the application but must support the communication module. The communication module includes key agreement and data encryption. The key agreement uses a scheme based on ECDSA and ECDH. Data encryption adopts a symmetric encryption scheme that mixes AES and RC4, and optimizes the speed of data encryption by taking advantage of the performance differences between AES and RC4 on data of different sizes.

### 3.2. Message Definition

This solution is based on the Diameter basic protocol, adopts part of the Diameter extension protocol, and adds custom messages as the supported messages of the IoT authentication protocol. Custom messages include Password-Update-Request, Key-Agreement-Request, and Key-Agreement-Answer, which are used for the key management functions of the authentication protocol. The rest of the messages are defined by the Diameter basic protocol and extended application protocol, which are used for authentication, authorization, and accounting functions.

The descriptions of the Diameter protocol messages and the custom messages used in this solution are shown in Table 1.

The command code of AA-Request (AAR) is 265, and the message flag bit “*R*” is set, indicating an authentication/authorization request. Among them, the user-name AVP and some other AVPs used for authentication need to be attached to the message for authentication and authorization. For example, the generated authentication element needs to be attached to the AAR and sent to the platform.

Re-Auth-Request (RAR) is set to 258 with the command code, indicating a reauthentication/-authorization request, and the message flag “*R*” is set. This command is sent by the platform to the node requesting reauthentication/-authorization of the node.

The command code of Re-Auth-Answer (RAA) is set to 258, and the message flag “*R*” is set to 0. This command is used to reply to the RAR message. Result-Code AVP must be present, indicating the processing result of the request message.

### 3.3. Key Agreement

This scheme designs a secure key agreement protocol that does not require a third-party trust center, and uses the ECDSA and the ECDH key agreement algorithm with less computational effort. Both nodes and platforms have public and private keys. When the ECDH algorithm is used for key negotiation, the random numbers generated by both parties are signed by the ECDSA and then sent to the other party. After the other party receives the data packet, the ECDSA is used for verification. If the verification is passed, the communication key is generated; otherwise, it is not generated.

The node requests permission to join the network and sends a Key-Agreement-Request (KAR) to the platform, which carries the node's ID and the signed ECDH public key *P*_*a*_.

(1) $$\begin{array}{c} P_{a}=P_{ec\text{d}h}R_{a}+PU_{a}•P_{ec\text{d}sa}. \end{array}$$

After the platform receives the KAR message, it uses the stored public key *PU*_*a*_ and *ecdsa* of the node to verify the validity of the *P*_*a*_ signature. If the verification is successful, a Key-Agreement-Request (KAR) is sent to the node, which carries the public key *P*_*b*_ signed with the private key of platform *b* to the DH public key *PU*_*b*_, and the Result-Code AVP is set to SUCCESS. In addition, according to *PU*_*a*_, *ecdh*, and *PR*_*b*_, ecdh generates a common symmetric key KE. If the verification fails, KAA is sent and the Result-Code AVP is failed.

(2) $$\begin{array}{c} P_{b}=P_{ec\text{d}h}U_{b}•\left(1-PR_{b}\right). \end{array}$$

**Figure 2:**
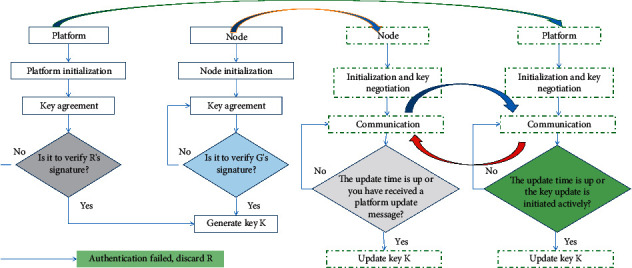
Node and platform key negotiation process.

After the node receives the KAA, it verifies the platform's signature. If the verification is successful, a common symmetric key KE is generated based on *PU*_*b*_ and *PR*_*a*_. If validation fails, the message is discarded. The two parties negotiate the symmetric key KE used for communication encryption according to the ECDH algorithm. The key negotiation process is shown in Figure 2.

In the initialization of nodes and platforms, the ECDSA and the ECDH algorithm need to be initialized. At the same time, some optimization can be made for a large number of elliptic multiplication operations. The parameters of the elliptic curve are consistent, and a set of values can be obtained for *G* in advance as a cache. When the node and platform need to perform elliptic multiplication with ECDSA and ECDH algorithms, they can directly get what they want from the cache or get the intermediate value to save computation.

Nodes have their own private key and public key, and nodes with the same access point can be selected to have the same private key and public key to reduce the complexity of the platform for node management. Among them, the node needs to sign its own random number, and the platform also needs to sign its own random number. Therefore, the two-way verification between the platform and the node is realized, and the security of the key negotiation process is guaranteed without the need for a third-party trusted center (CA).

### 3.4. Key Establishment of KMSBQF Scheme

Considering that the focus of this section is to analyze the quadratic orthogonal diagonalization characteristics when applied to WSN key management in terms of network security, for ease of discussion, the key management scheme is based on the following assumptions.

It is assumed that the network is homogeneous and static; that is, all nodes in the network are identical in software and hardware configuration, and once deployed, there will be no position movement, where the network size is *N*, and there are two types of nodes.

It is assumed that the base station BS is equipped with sufficient software and hardware resources, and its signal transmission range can cover the entire network deployment area by being equipped with high-power wireless signal transmitters and undertakes the central task of the entire network key distribution. Before network deployment, the base station generates an *n*-ary homogeneous quadratic polynomial key pool.

Then assign a quadratic polynomial to each ordinary node, collect and analyze the information sent by ordinary nodes, detect damaged or captured nodes, store the ID numbers of all ordinary nodes, and finally calculate the relationship between ordinary nodes.

Let the session key between nodes *m* and *a* be as follows:

(3) $$\begin{array}{c} K_{ma}=h\left(F\right)•\left(1+h\left(B\right)\right). \end{array}$$

Because the normalized diagonal matrix is orthogonal and the computation between the diagonal matrices is commutative (BF = FB),

(4) $$\begin{array}{c} K_{am}=h\left(F\right)•\left(1+h\left(B\right)\right)=h\left(B\right)•\left(1+h\left(F\right)\right)=K_{ma} \end{array}$$

shows that nodes *a* and *m* get the unique session key between them.

Assuming that nodes *a* and *f* are not adjacent, if the communication between nodes *a* and *f* is to be realized, first node *a* encrypts the neighbor list through the session key, realizes the exchange of its neighbor list with the neighbor node, and finds that the node *f* is adjacent to the neighbor node *m*.

While forwarding the information, the node *m* does not know the session content *M*, and this method ensures the security of the information during this process. In the same way, nodes exchange key information with each other, and then node *a* can communicate with any node in the network; that is, the connectivity rate of the network is 1.

### 3.5. Key Update

When a platform communicates with a node using network initialization or when a node joins the platform to negotiate a key, the security risks keep increasing. The platform needs to constantly evaluate the security of the entire platform. After a certain period of time or when the platform judges that the network is untrustworthy, it needs to perform a key update operation on all nodes in the network, and the nodes and the platform regenerate keys. The main problems here are as follows:

1. Due to the large number of nodes on the IoT platform, if the platform performs key update for nodes one by one, such as key renegotiation or key redistribution, this will have a great impact on the performance of the entire platform and will increase the platform's response time to nodes.
2. If the platform detects that the security of the network is reduced, it will trigger a key update. Maybe at this time, the key of the node has been analyzed. How to quickly update the key, while the platform triggers the node to update the key concurrently, becomes particularly important.

This section will introduce a key update scheme based on HASH function and time function. The platform broadcasts the message of updating the key, all nodes update their own key spontaneously, and the platform updates the key communicated with the node. In this way, the nodes are allowed to update their own keys concurrently, which solves the problem of network efficiency and node update efficiency caused by updating node keys one by one.

The principle of generating a new key by both parties is as follows: the platform broadcasts the information, and the node updates the key based on the timestamp. Since not all IoT nodes have time modules, the platform broadcast key information needs to carry timestamp information. The new key generation formula for nodes and platforms is as follows:

(5) $$\begin{array}{c} K_{N}=H\left(TS\right)|\left[H\left(K_{O}\right)-1\right]. \end{array}$$

Among them, *K*_*N*_ is the new key and, *K*_*O*_ is the old key.

The encryption keys of AES and RC4 are selected as 128 bits, and the SHA-1 hash function is used to obtain a 64-bit result from the operation of the old key. This solution can also customize a more flexible key update and can flexibly choose the parameters of the key update. The number of bits occupied by key and timestamp in the new key can be less than 64 bits. Insufficient bits are filled with 0. In this way, when the key is broken and the update algorithm is analyzed by the attacker, the parameters can be flexibly adjusted to ensure the security of the entire key management.

### 3.6. Certification Scheme

This scheme is an authentication scheme based on feature extraction, which ensures the security of authentication through feature extraction, and adopts the fusion of various authentication elements.

Nodes compose data packet AA-Request (AAR) message. Encrypt with the key KE obtained through key negotiation: if the message is larger than 100 bytes, use the RC4 encryption algorithm; if the message is less than or equal to 100 bytes, use the AES encryption algorithm. It consists of a data packet (AAAC) whose Command Class is COMMAND_CLASS_DIAMETER_AUTH (CCDA) and command is AAA, and is sent to the platform.

The platform receives AAAC and unpacks it to get AA-Request. Decrypt with KE to get AAR. Generate a 4-byte random number *R*, form an AA-Answer (AAA), and encrypt it with KE. Form the Z-Wave packet AAA command.

The platform compares the authentication element sent by the node. If they are the same, the authentication is successful, and the platform sends AAA to the node with Result-Code set to TRUE. If they are not the same, the authentication fails, and the platform sends AAA to the node, where the Result-Code is set to FALSE.

## 4. Results and Analysis

### 4.1. Anti-Capture Analysis

In the initial stage of the network, the KMSBQF scheme in this paper stores an independent and unique *n*-ary quadratic form and key information in any node. First, it breaks the conventional method and applies multivariate asymmetric polynomials to generate session keys, creating a polynomial-based predistribution scheme.

It breaks through the idea of Eschenauer–Gligor scheme single node storing multiple polynomials to improve the key sharing rate and q-composite scheme to improve the threshold of secure session. On the one hand, each node only stores a unique quadratic type and generates an independent and unique session key with each neighbor node. On the other hand, the different quadratic forms stored by each node cannot be deciphered by finding nodes containing the same quadratic form.

Since each quadratic is independent, it cannot be helped by capturing other nodes. In addition, as long as the dimension *n* of the quadratic matrix is slightly changed, the difficulty of cracking will increase greatly, which is much more difficult than cracking the predistribution scheme of the binary t-order symmetric polynomial key pool.

**Figure 3:**
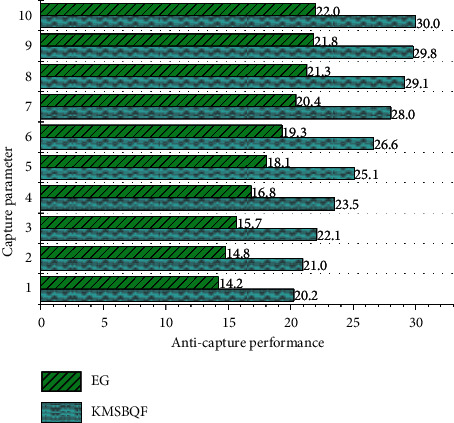
Anti-capture analysis, *n* = 10.

**Figure 4:**
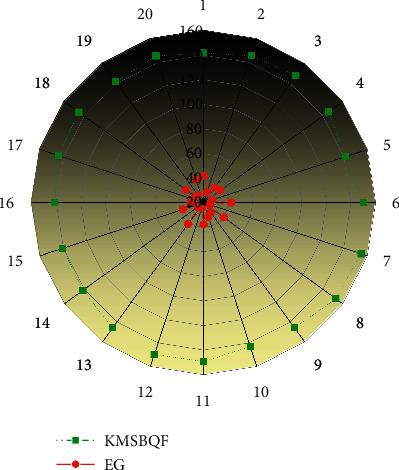
Anti-capture analysis, *n* = 20.

Assume that the parameter *n* is the order of the bivariate symmetric polynomial in the Eschenauer–Gligor scheme, that is, the bivariate n-degree symmetric polynomial, and *n* also represents the number *n* of the quadratic polynomial variables of the KMSBQF scheme in this paper. The Gligor scheme is difficult to resist t-collusion attack and to crack *f*(*xn*) in the KMSBQF scheme of this paper; it needs to crack the *n*(*n* + 1)/2 parameters of the symmetric matrix *A*. A comparison of the anti-trapping properties of the two schemes is shown in Figures 3 and 4.

With the increase of the capture parameter *n*, the anti-capture property of the Eschenauer–Gligor scheme changes proportionally, and the change is relatively gentle. As long as the enemy captures the same proportion of nodes, it is possible to threaten the network.

In the KMSBQF scheme, it is assumed that node *a* is captured. Since the key information (including quadratic) of the neighbor nodes used to calculate the session key and the Kpub communicated with the base station has been deleted, the attacker can only know that node *a* communicates with it. The session key between nodes (mainly neighbor nodes) and the key information of the nodes that are not involved in its communication are not known and will not affect the secure communication between other uncaptured nodes.

Even if the captured node *a* leaks the quadratic information stored by itself during the session key negotiation stage, since the authentication information stored by node *a* is processed by the one-way hash function, it is impossible for an attacker to crack the complete information.

At the same time, when the base station detects that node *a* is captured, it will broadcast to the entire network the fact that *a* will be deleted, stop all communication with *a*, delete all session keys of *a*, and also update the keys of all nodes adjacent to *a*, thereby improving the ability of the network to resist node capture attacks and ensuring the secure communication of the network.

Each authentication requires a certain amount of calculation, and the attacker can send a large number of messages, which can consume the energy of *a*, and then achieve the purpose of attacking the node. This scheme can resist such attacks. On the one hand, the attacker does not have the public key Kpub preassigned by the base station and has not established a neighbor list at the base station, so the attacker cannot escape the detection of the pseudo node of the base station. On the other hand, the attacker cannot calculate the session key without the method of two-factor authentication, so it is impossible to encrypt the session with the key.

### 4.2. Connectivity Analysis

In the KMSBQF scheme in this paper, any node in the network and its neighbor nodes can exchange key information, verify the identity of the node, and calculate the session key of both parties, thereby establishing a communication connection. Even nonadjacent nodes can obtain a communication link by exchanging neighbor lists and then forward information through intermediate nodes in the link to realize a conversation between two nodes. Therefore, communication can be achieved between any nodes in the network; that is, the connectivity rate of the network in this scheme is 1.

However, there is a probability problem in the Eschenauer–Gligor scheme; that is, there may be some nodes that do not share keys with surrounding neighbor nodes, and there is no key path, so the connectivity of the network cannot be guaranteed. The connectivity ratio of the Eschenauer–Gligor scheme is as follows:

(6) $$\begin{array}{c} P_{EG}=\frac{\left(2p-k\right)!}{p!\left(p-2k\right)!}-\frac{\left(p-2k\right)!}{p!\left(2p-k\right)!}, \end{array}$$

where *P* is the probability that adjacent nodes share a key and *k* is the size of the key chain. Therefore, the factors that affect the network connectivity are the deployment density of the network, the condition of the target area, the size of the key pool *S*, and the size *k* of the node key chain. The greater the *k*/*S*, the greater the probability that there is a shared key between neighboring nodes. However, with larger *k*/*S*, the network security will become vulnerable, because too large *k* will occupy too much resources of the node, and if *S* is too small, it is easy for an attacker to obtain most of the keys in the key pool by capturing a small number of nodes, thereby endangering the cyber security.

Compared with the Eschenauer–Gligor scheme, the q-composite scheme has better survivability of the network, and the capture of a small number of nodes will not affect the communication between other nodes in the network. The connectivity ratio of the q-composite scheme is as follows:

(7) $$\begin{array}{c} P_{q}=1-\prod_{i=1}^{q}\frac{\left(\begin{array}{c} S-i\\ k-i \end{array}\right)\left(\begin{array}{c} 2S-i\\ 2\left(k-i\right) \end{array}\right)\left(\begin{array}{c} S\\ k-i \end{array}\right)}{\left(\begin{array}{c} S-2i\\ 2\left(k-i\right) \end{array}\right)\left(\begin{array}{c} S-i\\ k-i \end{array}\right)}. \end{array}$$

Among them, *S* is the size of the key pool, *k* is the size of the key chain, and *q* is the number of shared keys. However, if the probability of at least *q* shared keys between adjacent nodes in the network reaches the preset probability, the size of the entire key pool must be reduced and the overlap of shared keys between nodes must be increased, thus limiting the network's scalability. An attacker can obtain most of the keys in the key pool by capturing a small number of nodes.

**Figure 5:**
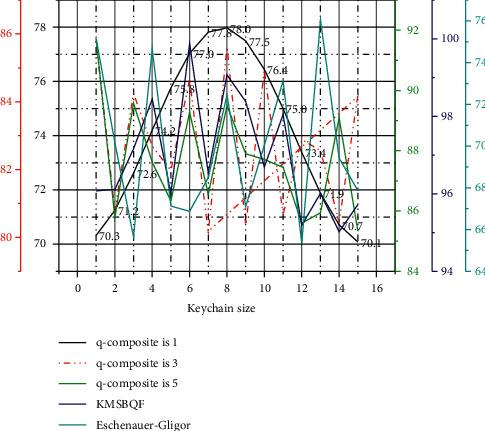
The network connectivity rate varies with the size of the key chain.

The simulation results are shown in Figure 5. The connectivity rate of the KMSBQF scheme is always 1, because its connectivity rate has nothing to do with the size of the key pool. As long as the quadratic type can be allocated, the connectivity of the network can be guaranteed to be 1; at the same time, the connectivity rate of the Eschenauer–Gligor scheme is significantly lower than that of other schemes. There are two schemes; the q-composite scheme will improve the connectivity rate as *q* increases; and both the q-composite scheme and the Eschenauer–Gligor scheme will improve the connectivity rate as the key chain becomes larger.

### 4.3. Overhead Analysis

**Figure 6:**
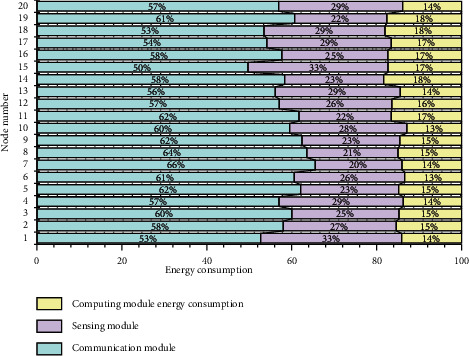
Energy consumption distribution of nodes.

When a security protocol is added to the wireless sensor network, 20% of it is consumed in the process of shared key discovery and establishment, and the energy consumed per 1 bit of data transmission is higher than that of the security algorithm. Therefore, the KMSBQF scheme in this paper increases some node overhead due to quadratic calculation, but it is realistic and acceptable for current sensor nodes, and the cost is worth the improvement of WSN security performance. In fact, the energy consumption of wireless sensor network nodes mainly lies in the communication module, and the energy consumption of the sensor module and the computing module is relatively small. The energy consumption distribution of nodes is shown in Figure 6.

In the initialization stage of the KMSBQF scheme, each node needs an encryption operation when sending secret information to the base station; in the key establishment stage, the node needs to perform a quadratic polynomial analysis to obtain a quadratic matrix. One linear equation system operation is used to solve the eigenvectors, one Gram–Schmidt orthogonalization operation is used to solve the orthogonal matrix, three hash operations are used to verify the identity, and one hash operation is used to form the session key. Although many operations are performed in the initialization phase, when the session key is established between nodes, only one encryption operation and one decryption operation are required each time, and the amount of computation is relatively small, so the computational overhead of this scheme is acceptable.

Under the premise of not considering the security problem, the calculation cost of the Eschenauer–Gligor scheme to establish the session key is the smallest. As long as the neighbor nodes find a common key identifier, the establishment of the session key can be completed. Accordingly, the computational overhead of the Eschenauer–Gligor scheme is negligible.

For the q-composite scheme, after discovering the shared key, it is determined that there are *t* shared keys with its own neighbor nodes, *t* > *q*; then, a one-way hash function can be used to establish the session key. The establishment of the session key is only performed by a hash operation, which also has a small computational overhead. However, in order to ensure the connectivity of the network with a certain probability, the size of the key pool must be reduced, and the degree of overlap between nodes must be increased. Due to the scalability of the network, an attacker can capture most of the keys in the key pool by capturing a small number of nodes, which also sacrifices the security of the network.

In the key establishment phase, a secret message is sent to the base station, informing the base station to store the list information, and a broadcast communication between neighbor nodes is required to generate a session key between the node and all neighbor nodes. In the Eschenauer–Gligor and q-composite schemes, the node needs to perform a broadcast during the key discovery stage, and when there is a shared key between two nodes, an information exchange is required to generate the session key. When there is no shared key, a communication link needs to be established through a third node or more intermediate nodes.

### 4.4. Personnel Information Management Test

**Figure 7:**
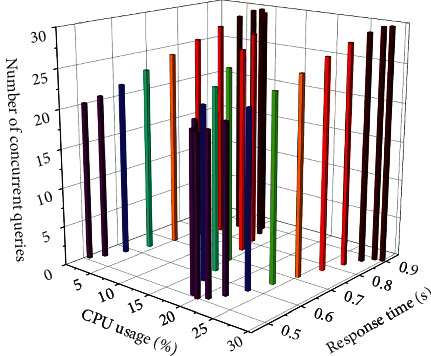
Business indicator test.

**Figure 8:**
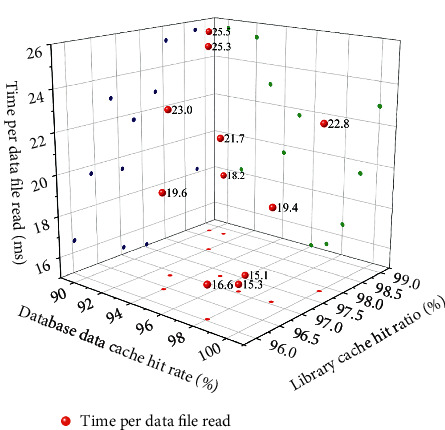
Database indicator test.

The host performance indicators are shown in Table 2. The purpose of the performance test is to verify whether the system meets the user's requirements and has high performance. The main content of the test includes the response time of the system and the concurrency of the system. The indicators of this system performance test are CPU utilization, memory consumption, disk, maximum concurrent users, memory usage, database indicators, response time, and concurrent query response time. The business indicators and database indicators are shown in Figures 7 and 8, respectively.

The main task of document testing is to check the documents in the system development process to verify the correctness of the documents. The main content of the document test is to test various documents in the system development phase and the user's product specification, such as testing whether the content of various documents in the development phase is accurate and complete, whether the language description is clear, whether the user's product specification is concise, whether the language is standardized, whether it conforms to the user's reading logic, and whether the system meets the user's needs.

## 5. Conclusion

Aiming at overcoming the security problems and deficiencies of the Z-Wave protocol, a Diameter-based IoT authentication protocol is introduced, which includes billing, authentication/authorization, and secure communication. The protocol comprehensively considers the characteristics of the node's computing power, energy consumption, and storage space. In addition to secure communication, which must be implemented, billing and authentication/authorization can be selected according to the characteristics of the application. Authentication and authorization are subject to the given client and server state machines. In terms of secure communication, different encryption communication schemes are introduced for different data. Aiming at solving the problem that the existing polynomial-based key predistribution management scheme is limited by the key sharing rate between the nodes and the network connectivity rate, a quadratic-based wireless sensor key management scheme is proposed. The scheme breaks through the existing idea of building a shared key with a binary t-degree symmetric polynomial, introduces a multivariate asymmetric quadratic polynomial, and uses the relationship between the quadratic eigenvalue and eigenvector to prove through analysis that the quadratic can be orthogonal. The node realizes identity authentication by exchanging key information and finally completes the establishment of an independent and unique session key with neighboring nodes. The security performance analysis and simulation results show that, compared with the existing key management schemes, the scheme has great improvements in anti-capture property, connectivity, scalability, communication overhead, and storage overhead. System testing is the most important link before the software is put into operation. It is an important way to ensure the quality of the software and improve the performance and efficiency of the software. Testers design a batch of test cases according to user demand analysis, development specifications, business processes, etc. and then use these test cases to test the program in order to find problems in the software running process, so as to find software defects in time. The most direct purpose of system testing is to find software errors as much as possible, to make as many errors as possible through limited time and number of tests, and to analyze the causes and trends of errors in order to better solve these errors. However, system testing is not only to find errors; the most important task of testing is to find the degree of matching between software and user requirements, and to deal with the problems found in time to improve the usability of the software.

## Figures and Tables

**Table 1:** Messages and their command codes.

Message name	Short name	Command code
Session-Termination-Request	STR	276
Password-Update-Request	PUR	475
Key-Agreement-Answer	KAA	487
AA-Request	AAR	265
AA-Answer	AAA	355
Accounting-Request	ACR	238

**Table 2:** Host performance indicators.

Indicator	Value
Disk	0.22
Maximum concurrent users	385
Memory usage	240 MB–570 MB
CPU utilization	1%–30%
Memory consumption	0.03

## Data Availability

The data used to support the findings of this study are available from the corresponding author upon request.
